# Editorial: Transparency Must Prevail

**DOI:** 10.1523/ENEURO.0300-17.2017

**Published:** 2017-09-06

**Authors:** Christophe Bernard

The theme of this year's Peer Review Week is transparency. During the last few decades, I believe that a lack of transparency in the review process has degraded the field of scientific publishing. Authors were frequently left confused and angry when conflicting reviews lead to a rejection or when their paper received an obscure rejection and was not reviewed at all. Some journals still use the infamous box “confidential remarks to the editor.” These practices led to a growing sense of dissatisfaction among all scientists, myself included.

Fortunately, times have changed. There is now a growing number of journals that have a more transparent peer-review system, and *eNeuro* is proud to be among these journals. At *eNeuro* the decisions and the reviews are based on facts that are communicated to the authors and there are no confidential remarks to the editor. What would be the point? If there is something wrong in the paper or a doubt about the work, it is conveyed directly to the authors. Also, because our editors at *eNeuro* are actively engaged in research in the field, they are well positioned to assess the validity of the reviewers’ comments. Just like the conclusions we draw from our own research, decisions at *eNeuro* are thoroughly justified and based on facts.

Inconsistency between reviews has also been a problem. When there are both positive and negative reviews, the final decision is often negative. Why? As authors, we rarely got a justification. Again, the solution is simple. One document–one voice–should be conveyed to the authors. *eLife* led the way. They set up a consultation process between reviewers and the reviewing editor (also an active research scientist), who work together until a consensus is reached. This consensual decision is then conveyed to the authors. This is a very good way to ensure transparency and restore confidence in the peer-review process. Reviewers and Reviewing Editor speak with one voice, nothing is hidden and the decision does not accompany contradictory suggestions for improving the paper. This is the solution that we have also adopted at *eNeuro*.

And it does work. How do we know? Since 2014, we have received less than a handful of appeals. This means that authors received enough facts to understand why their papers were rejected. Both authors and reviewers who have experienced the consultation-consensus process report how much they enjoy the procedure and its transparency and clarity. You can hear a few of our editors and an author discuss this process in our latest video ‘What if the Peer Review Process was Painless’ currently shown on *eNeuro’s* homepage.

Of course, transparency can (and should) be continually improved. Ideally, the names of the reviewers could be disclosed whether the paper is accepted or rejected. Although, this may pose problems, one being that reviewers may fear future retribution from unhappy authors. Going for full transparency, and agreeing on what that means, should be a collegial decision and involve discussions among all stakeholders and with many journals.

At *eNeuro*, we are striving to increase transparency, and we are constantly thinking about novel ways to improve it (e.g., our recent new policy regarding codes for computational neuroscience papers). We are about to begin an initiative to allow authors to “review” the reviewing process for their paper. Authors who submit with *eNeuro* will find a survey in their decision letter that allows them to provide us feedback. These responses will increase transparency by informing our reviewing editors and me how authors experience our reviewing process. We are here to help authors, and authors can help us to make a better journal. Stay tuned!

Cheers,

